# Supplementary material for: Loss of neuronal Miro1 disrupts mitophagy and induces hyperactivation of the integrated stress response
Source: EMBO J. 2021 Jun 21;40(14):e100715. doi: 10.15252/embj.2018100715 (PMC8280823; doi:10.15252/embj.2018100715)
Supplement: Supplementary file 1 — Appendix [file EMBJ-40-e100715-s007.pdf]

Table of contents:

- Appendix Table S1

- Appendix References

- Appendix Figure Legends

- Appendix Figures (S1 to S9)

Appendix Table S1: Lysine residues identified as potential sites for ubiquitination

In Wagner et al., 2012 work is performed from mouse tissue/cells

	Lysine (Human)	Lysine (Mouse)	Steady State	Mitochondrial Damage	Other	*In vitro* studies
Miro1	**K92**	K105	(Akimov et al., 2018)			
	**K107**	K120			(Boeing et al., 2016)[a]	
	**K153**	K166	(Wagner et al., 2011; Wagner et al., 2012)	(Sarraf et al., 2013)		(Kazlauskaite et al., 2014; Klosowiak et al., 2016)
	**K182**	K195	(Akimov et al., 2018; Wagner et al., 2012)			
	**K187**	K200	(Akimov et al., 2018; Mertins et al., 2013; Udeshi et al., 2013; Wagner et al., 2011; Wagner et al., 2012)	(Ordureau et al., 2015; Ordureau et al., 2018; Ordureau et al., 2014)	(Boeing et al., 2016)[a]; (Wu et al., 2015)[b]; (Povlsen et al., 2012)[a]	
	**K194**	K207	(Akimov et al., 2018; Wagner et al., 2012)	(Sarraf et al., 2013)		
	**K230**	K243	(Akimov et al., 2018)			(Kazlauskaite et al., 2014)
	**K235**	K248	(Akimov et al., 2018)	(Sarraf et al., 2013)		(Kazlauskaite et al., 2014; Klosowiak et al., 2016)
	**K249**	(K to R)		(Ordureau et al., 2014)		
	**K330**	K343	(Akimov et al., 2018)			(Kazlauskaite et al., 2014)
	**K427**	K440	(Akimov et al., 2018)			
	**K512**	K525	(Udeshi et al., 2013; Wagner et al., 2012)	(Ordureau et al., 2018)		
	**K535**	K548	(Akimov et al., 2018)			
	**K572**	K585		(Ordureau et al., 2018; Ordureau et al., 2014; Sarraf et al., 2013)		(Kazlauskaite et al., 2014; Klosowiak et al., 2016)

[a] U.V. irradiation

[b] Treatment with HDAC inhibitors

Appendix References

Akimov, V., Barrio-Hernandez, I., Hansen, S.V.F., Hallenborg, P., Pedersen, A.K., Bekker-Jensen, D.B., Puglia, M., Christensen, S.D.K., Vanselow, J.T., Nielsen, M.M.*, et al.* (2018). UbiSite approach for comprehensive mapping of lysine and N-terminal ubiquitination sites. Nat Struct Mol Biol *25*, 631-640.

Boeing, S., Williamson, L., Encheva, V., Gori, I., Saunders, R.E., Instrell, R., Aygun, O., Rodriguez-Martinez, M., Weems, J.C., Kelly, G.P.*, et al.* (2016). Multiomic Analysis of the UV-Induced DNA Damage Response. Cell reports *15*, 1597-1610.

Kazlauskaite, A., Kelly, V., Johnson, C., Baillie, C., Hastie, C.J., Peggie, M., Macartney, T., Woodroof, H.I., Alessi, D.R., Pedrioli, P.G.*, et al.* (2014). Phosphorylation of Parkin at Serine65 is essential for activation: elaboration of a Miro1 substrate-based assay of Parkin E3 ligase activity. Open Biol *4*, 130213.

Klosowiak, J.L., Park, S., Smith, K.P., French, M.E., Focia, P.J., Freymann, D.M., and Rice, S.E. (2016). Structural insights into Parkin substrate lysine targeting from minimal Miro substrates. Sci Rep *6*, 33019.

Mertins, P., Qiao, J.W., Patel, J., Udeshi, N.D., Clauser, K.R., Mani, D.R., Burgess, M.W., Gillette, M.A., Jaffe, J.D., and Carr, S.A. (2013). Integrated proteomic analysis of post-translational modifications by serial enrichment. Nat Methods *10*, 634-637.

Ordureau, A., Heo, J.M., Duda, D.M., Paulo, J.A., Olszewski, J.L., Yanishevski, D., Rinehart, J., Schulman, B.A., and Harper, J.W. (2015). Defining roles of PARKIN and ubiquitin phosphorylation by PINK1 in mitochondrial quality control using a ubiquitin replacement strategy. Proceedings of the National Academy of Sciences of the United States of America *112*, 6637-6642.

Ordureau, A., Paulo, J.A., Zhang, W., Ahfeldt, T., Zhang, J., Cohn, E.F., Hou, Z., Heo, J.M., Rubin, L.L., Sidhu, S.S.*, et al.* (2018). Dynamics of PARKIN-Dependent Mitochondrial Ubiquitylation in Induced Neurons and Model Systems Revealed by Digital Snapshot Proteomics. Mol Cell *70*, 211-227 e218.

Ordureau, A., Sarraf, S.A., Duda, D.M., Heo, J.M., Jedrychowski, M.P., Sviderskiy, V.O., Olszewski, J.L., Koerber, J.T., Xie, T., Beausoleil, S.A.*, et al.* (2014). Quantitative proteomics reveal a feedforward mechanism for mitochondrial PARKIN translocation and ubiquitin chain synthesis. Mol Cell *56*, 360-375.

Povlsen, L.K., Beli, P., Wagner, S.A., Poulsen, S.L., Sylvestersen, K.B., Poulsen, J.W., Nielsen, M.L., Bekker-Jensen, S., Mailand, N., and Choudhary, C. (2012). Systems-wide analysis of ubiquitylation dynamics reveals a key role for PAF15 ubiquitylation in DNA-damage bypass. Nature cell biology *14*, 1089-1098.

Sarraf, S.A., Raman, M., Guarani-Pereira, V., Sowa, M.E., Huttlin, E.L., Gygi, S.P., and Harper, J.W. (2013). Landscape of the PARKIN-dependent ubiquitylome in response to mitochondrial depolarization. Nature *496*, 372-376.

Udeshi, N.D., Svinkina, T., Mertins, P., Kuhn, E., Mani, D.R., Qiao, J.W., and Carr, S.A. (2013). Refined preparation and use of anti-diglycine remnant (K-epsilon-GG) antibody enables routine quantification of 10,000s of ubiquitination sites in single proteomics experiments. Mol Cell Proteomics *12*, 825-831.

Wagner, S.A., Beli, P., Weinert, B.T., Nielsen, M.L., Cox, J., Mann, M., and Choudhary, C. (2011). A proteome-wide, quantitative survey of in vivo ubiquitylation sites reveals widespread regulatory roles. Mol Cell Proteomics *10*, M111 013284.

Wagner, S.A., Beli, P., Weinert, B.T., Scholz, C., Kelstrup, C.D., Young, C., Nielsen, M.L., Olsen, J.V., Brakebusch, C., and Choudhary, C. (2012). Proteomic analyses reveal divergent ubiquitylation site patterns in murine tissues. Mol Cell Proteomics *11*, 1578-1585.

Wu, Q., Cheng, Z., Zhu, J., Xu, W., Peng, X., Chen, C., Li, W., Wang, F., Cao, L., Yi, X., *et al.* (2015). Suberoylanilide hydroxamic acid treatment reveals crosstalks among proteome, ubiquitylome and acetylome in non-small cell lung cancer A549 cell line. Sci Rep *5*, 9520.

Appendix Figure Legends

Appendix Figure S1:

(A) Representative images of WT and Myo19^{KO} RpE1 cells transfected with ^{YFP}Parkin (green) and treated with FCCP (10 μM) for 1 hour to induce mitophagy. Cells were immunostained with anti-Tom20 (magenta) to reveal the mitochondrial network. (B) Quantification of the different stages of mitophagy as defined in Figure 1 in WT and Myo19^{KO} RpE1 cells treated with FCCP (10 μM) for the times specified. RpE1 cells appeared to be more sensitive to mitophagy induction by FCCP treatment as half of the cells present the total translocation of Parkin to the mitochondrial network as soon as at 6 hours of treatment. However, Myo19 deletion does not appear to have any effect on the dynamics of Parkin recruitment to damaged mitochondria. Data collected from 3 independent experiments (n=3; one way ANOVA with Dunnett post test).

Error bars represent s.e.m. Significance: (ns = not significant)

Appendix Figure S2:

(A) Quantification of protein levels of all Miro1 ubiquitination mutants shown in Figure 2C. While there is a small reduction in the expression of $Miro1^{K182R}$ and $Miro1^{K572R}$, the mutant constructs used throughout the study, $Miro1^{5R}$ and $Miro1^{allR}$, do not express at significantly different levels to $Miro1^{WT}$ (data collected from 3 different expression experiments (n=3); One way ANOVA and Newman-Keuls *post hoc* analysis). (B) Quantification of Miro1 expression levels in the ubiquitination assays in ^{Flag}Parkin overexpressing SH-SY5Y cells transfected with $Miro1^{WT}$, $Miro1^{5R}$ or $Miro1^{allR}$ or $Miro1^{R572K}$ constructs and treated with FCCP (10 μM) for 6 hours (n=4 independent experiments; ANOVA with Sidak's *post hoc* test for all comparisons).

Error bars represent s.e.m. Significance: *$p<0.05$, **$p<0.01$ and ***$p<0.001$.

Appendix Figure S3:

(A) Representative image and details of $Miro^{DKO}$ cells co-transfected with $^{myc}Miro1^{WT}$ and GFP as a reporter of the transfection and stained with TMRM to measure and compare mitochondrial membrane potential in control, untransfected (i) and Miro1 mutant constructs transfected cells (ii) at the same time. (B) Quantification of TMRM intensity for the conditions specified. Ddata collected from 3 different experiments (n=3; Student t-test).

Error bars represent s.e.m. Significance: (ns = not significant)

Appendix Figure S4:

(A) Representative images showing Miro^{DKO} cells transfected with the specified constructs (top panels) growing in “Y” shaped micropatterns. MitoTracker staining (lower panels) reveal mitochondrial distribution in these cells. (B) The cumulative distribution of mitochondrial signal or Mitochondrial Probability Map (MPM) is plotted for the different genotypes. A displacement to the right compared to Miro^{DKO} control cells indicates that mitochondrial signal is redistributed towards the periphery of the cell. The grey dotted line represents the theoretical distribution of a homogeneously distributed signal. (C) Graph showing the calculated Mito^{95} values (95th percentile) which represent the distance from the cell centre at which 95% of the mitochondrial signal is found. Analysis in B and C were performed from at least 3 independent experiments (number of experiments: WT 4, Miro^{DKO} 4, Miro^{DKO} + Miro1^{WT} 4, Miro^{DKO} + Miro1^{5R} 4, and Miro^{DKO} + Miro1^{allR} 3; ANOVA and post hoc Newman-Keuls) with at least 20 cells where analysed per genotype and experiment. Two different cell lines were used per genotype.

Error bars represent s.e.m. Significance: *$p<0.05$, **$p<0.01$ and ***$p<0.001$.

Appendix Figure S5:

Cytotoxicity of known mitochondrial depolarising agents on primary neuronal cultures. (A) Example confocal images of cultures stained with DAPI (all nuclei) and PI (nuclei of dead cells) after 3 hours of drug treatment. Blue arrow indicates a viable cell. Red arrow indicates a dead cell (scale bars =100 µm). (B) Quantification of the cytotoxicity of each drug, calculated as the number of dead (PI+) cells as a percentage of total (DAPI+) cells. Data collected from 4 different neuronal preparations (n=8 different replicates belonging to 8 different embryos; one-way ANOVA).

Error bars represent s.e.m. Significance: *p<0.05, **p<0.01 and ***p<0.001.

Appendix Figure S6:

Loss of Miro1 delays Parkin recruitment to damaged mitochondria in neurons. (A) Uncropped example confocal (63x) images of WT and Miro1^{KO} neurons expressing MtdsRed and ^{YFP}Parkin following valinomycin treatment (scale bars = 10 µm). (B) Example cropped and straightened confocal images of dendrites from WT and Miro1^{KO} neurons expressing MtdsRed and ^{YFP}Parkin following valinomycin treatment (Scale bars =5 µm).

Appendix Figure S7:

(A) Representative confocal images of the soma of $PINK1^{KO}$ cortical neurons expressing WT and mutant forms of Miro1, ^{YFP}Parkin and MtDsRed without valinomycin treatment (scale bars = 10 µm). (B) Representative confocal images of the soma of $PINK1^{KO}$ cortical neurons expressing WT and mutant forms of Miro1, ^{YFP}Parkin and MtDsRed after 5 hours of valinomycin treatment (scale bars = 10 µm).

Appendix Figure S8:

(A-B) Quantification of Mitofusin1 and Mitofusin2 mRNA levels in 12 month old WT and Miro1^{CKO} hippocampus using GAPDH (A) or HPRT (B) as housekeeping genes showing no difference in the expression of either Mitofusin1 or Mitofusin2 in aged Miro1^{CKO} animals. Quantitative PCRs were performed 3 different times with all samples processed in triplicate from 4 different animals for each genotype (n=4; Student's t-test). (C) Representative western blot images of lysates from WT and Miro1^{KO} cortical neurons immunoprecipitated with an anti-Mfn2 antibody and immunoblotted with the antibodies stated. (D) Quantification of ubiquitin band intensity (60-250 kDa) normalised to Mfn2 band intensity from WT and Miro1^{KO} cortical neurons (n=2 WT and n=4 Miro1^{KO} neuronal preparations; unpaired t-test).

Error bars represent s.e.m. Significance: *$p<0.05$, **$p<0.01$ and ***$p<0.001$.

Appendix Figure S9:

(A) Schematic depicting the mouse lines used to generated control (Miro1(Δ/+)) and conditional knockout animals (Miro1(Δ/Δ)) with labelled mitochondria in only the cells of interest. (B) High resolution AiryScan images of MitoDendra expression in WT and Miro1^{CKO} projection neurons in cortex. The mitochondrial network can be observed in the somas of WT neurons while they appear missdistributed and collapsed onto big and swollen giant mitochondria in Miro1^{CKO} somas. These large mitochondria present enhanced ubiquitin signaling (arrowhead) in a subset of cells (C).

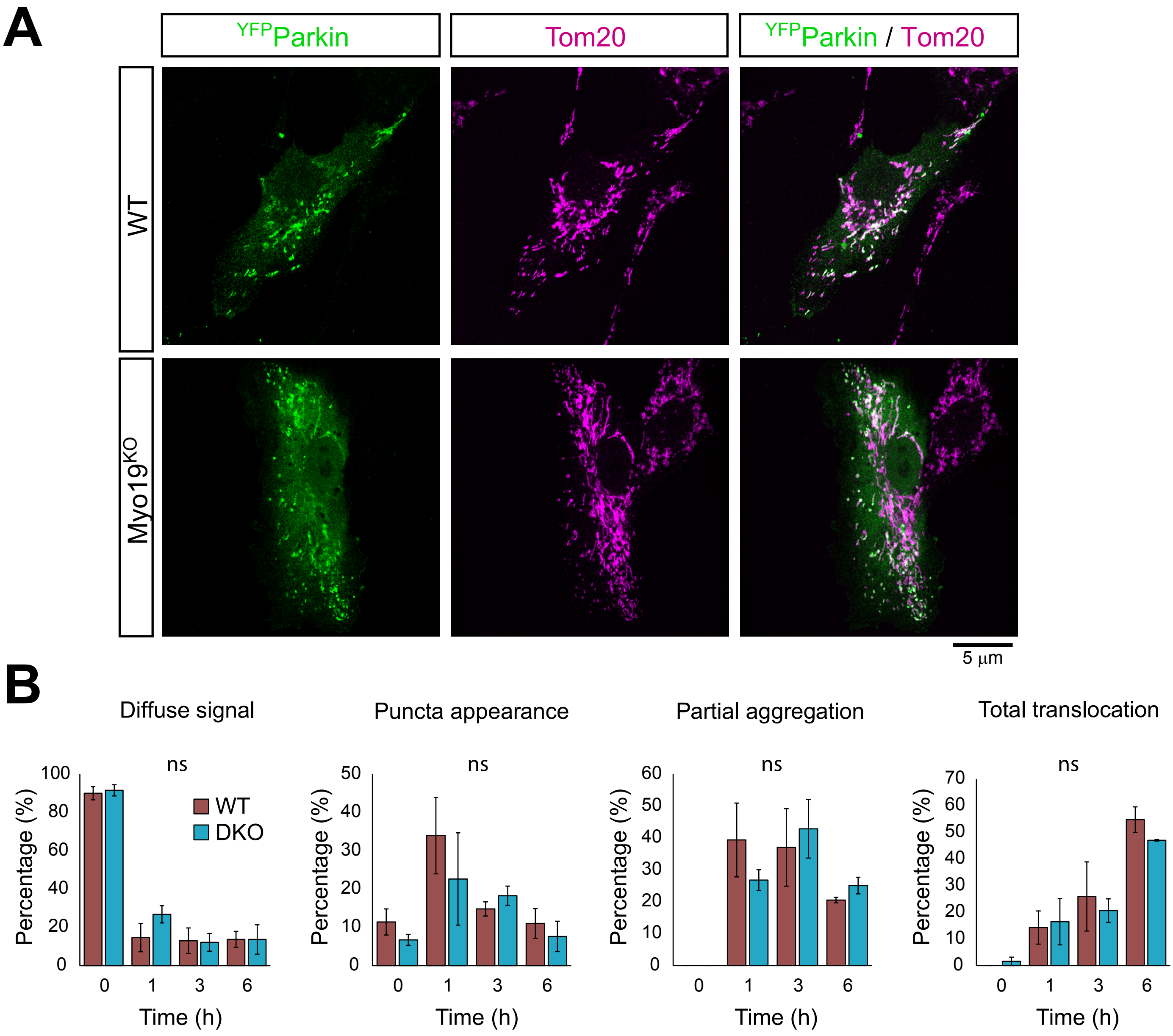

Appendix Figure S1

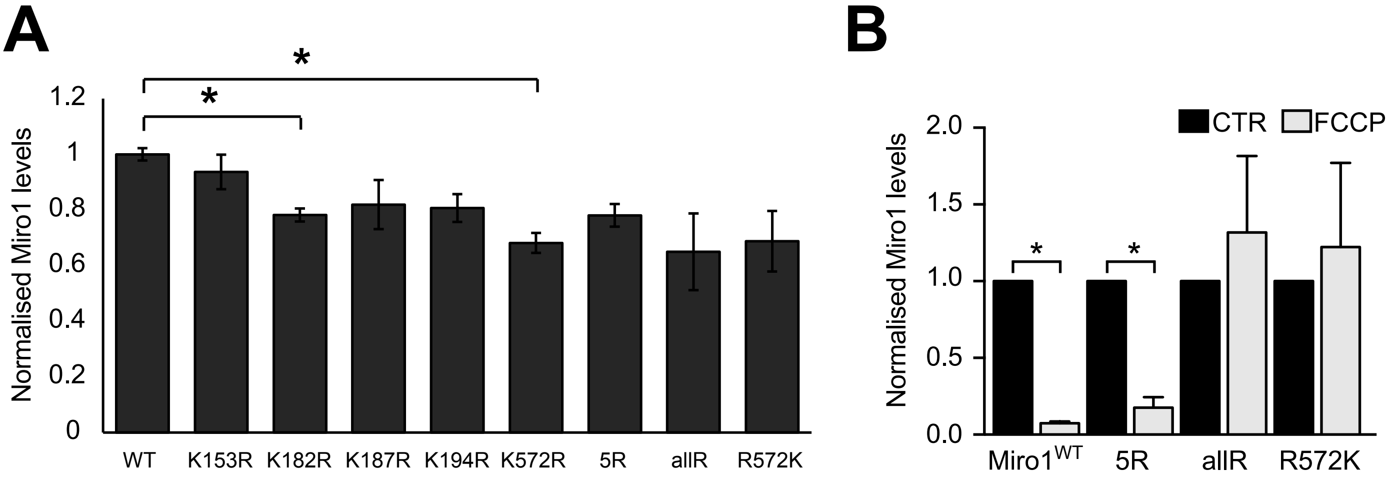

Appendix Figure S2

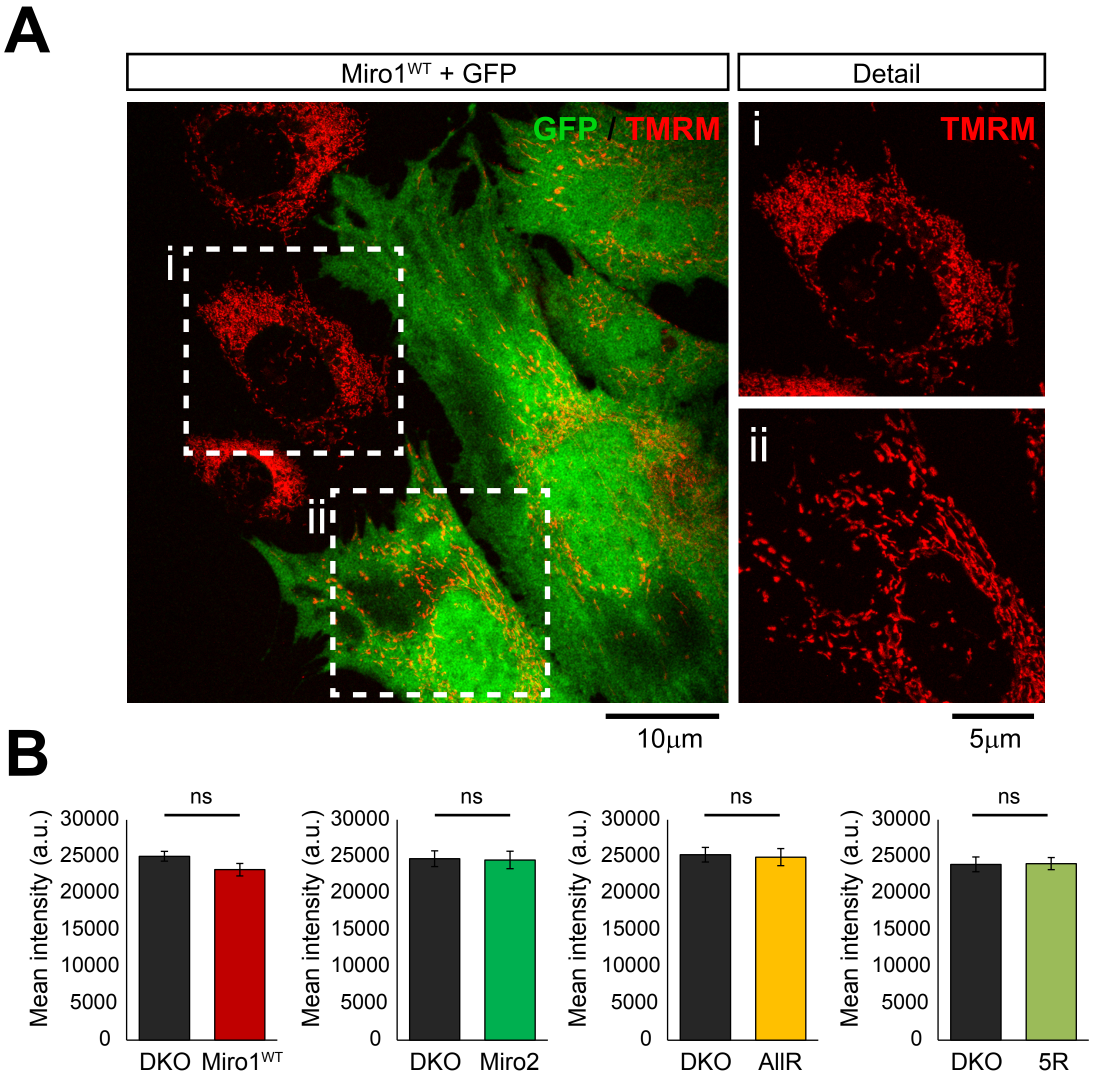

Appendix Figure S3

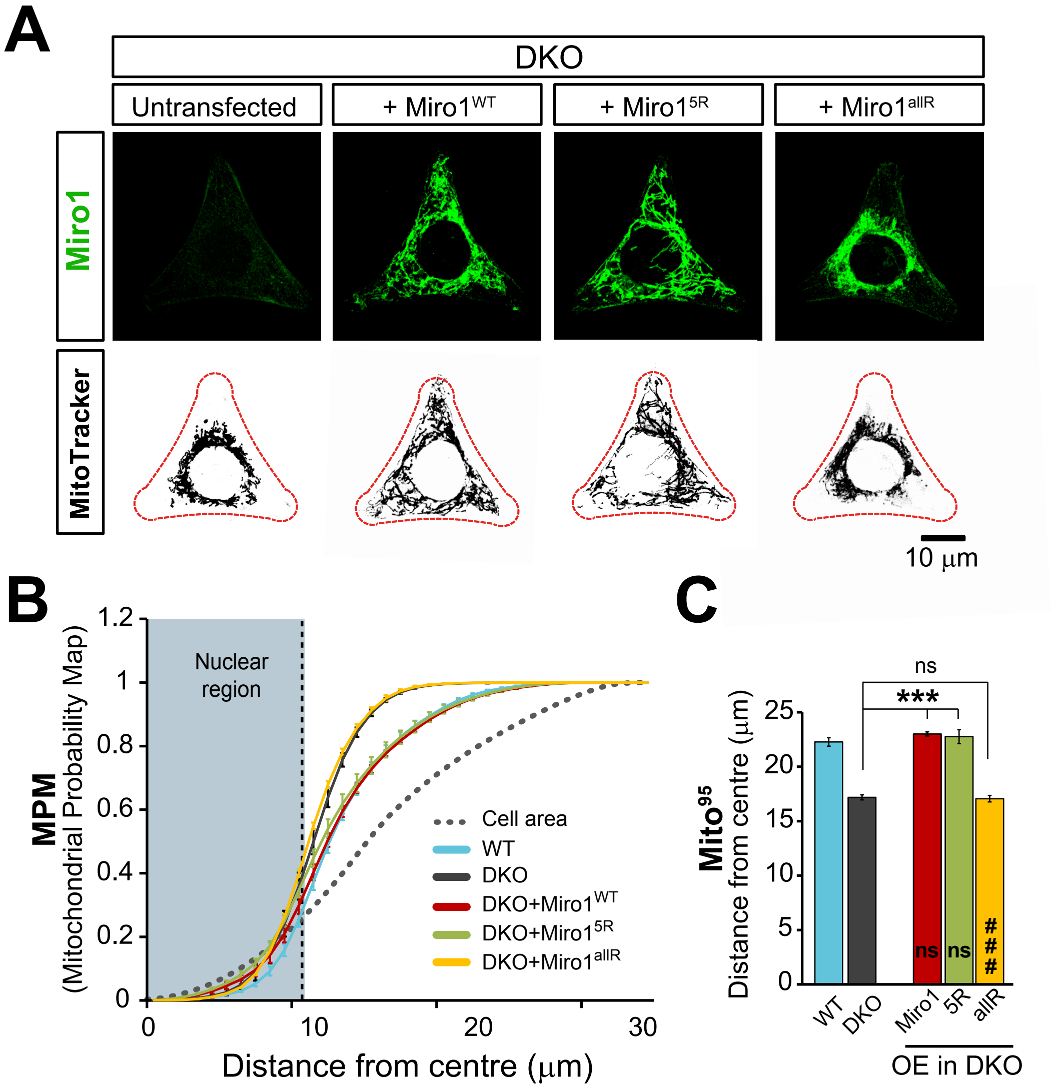

Appendix Figure S4

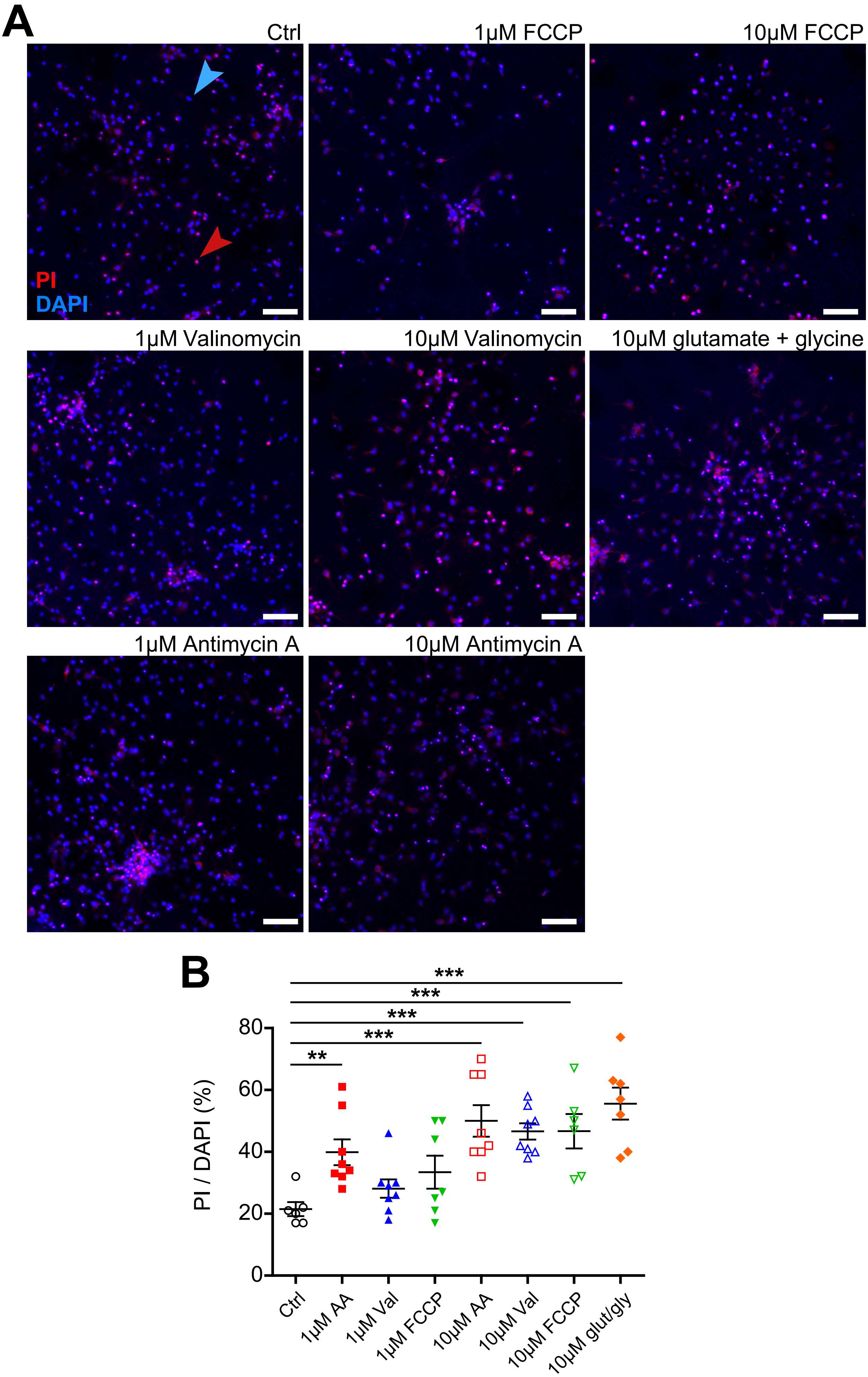

Appendix Figure S5

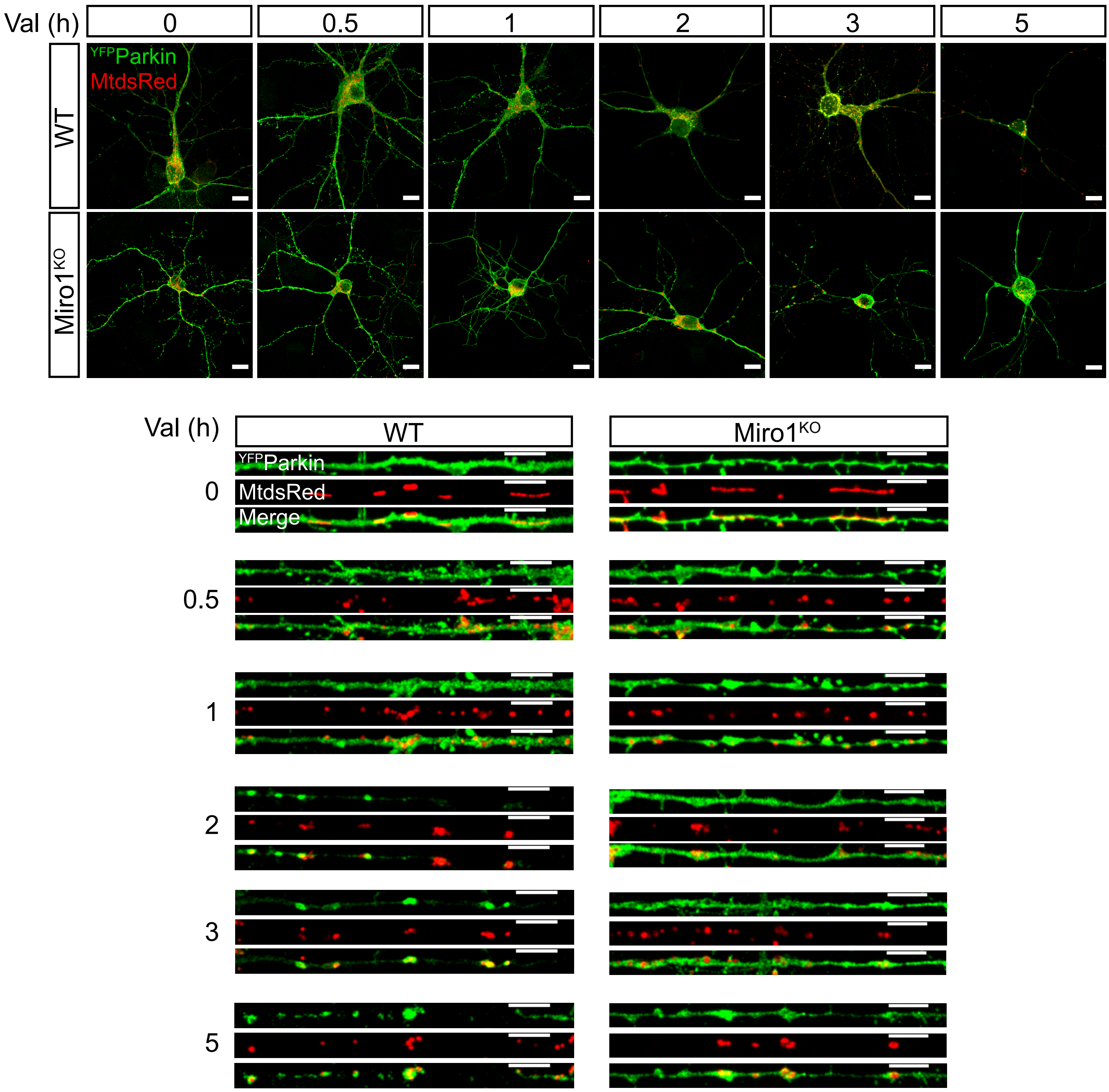

Appendix Figure S6

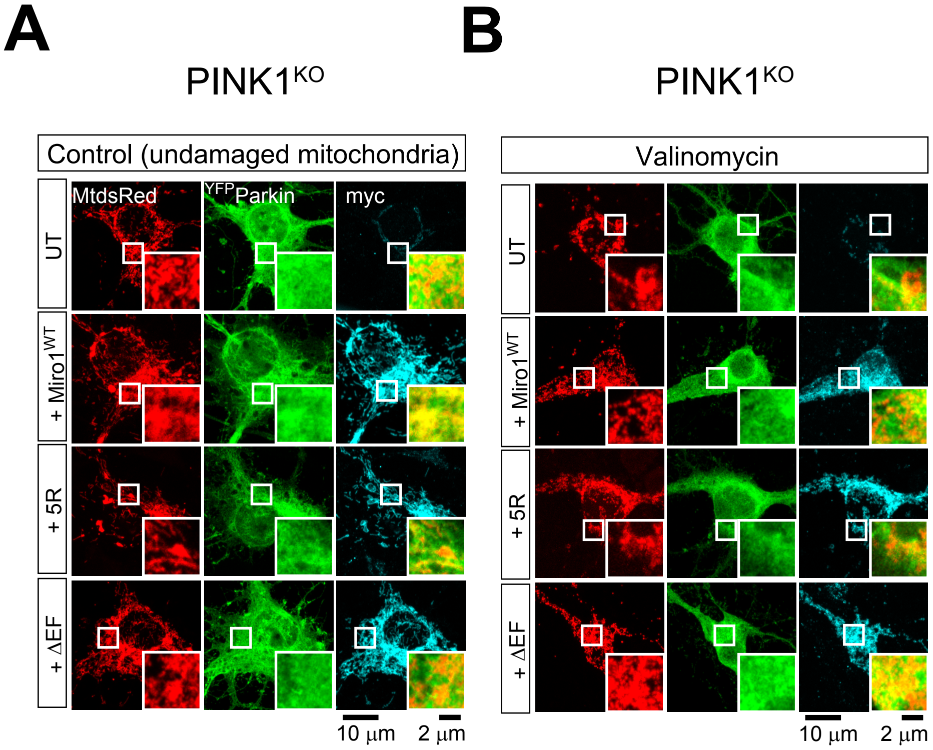

Appendix Figure S7

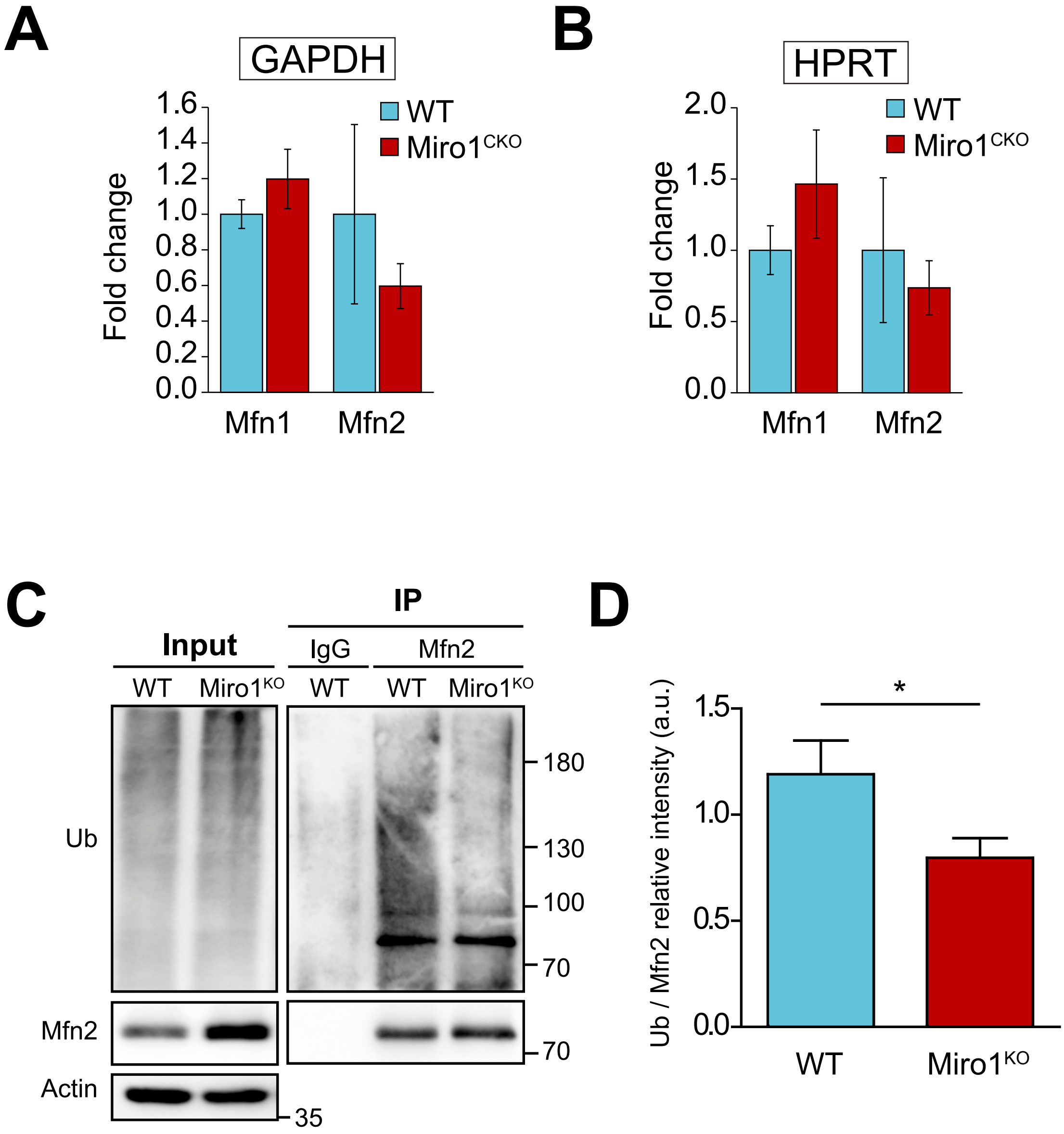

Appendix Figure S8

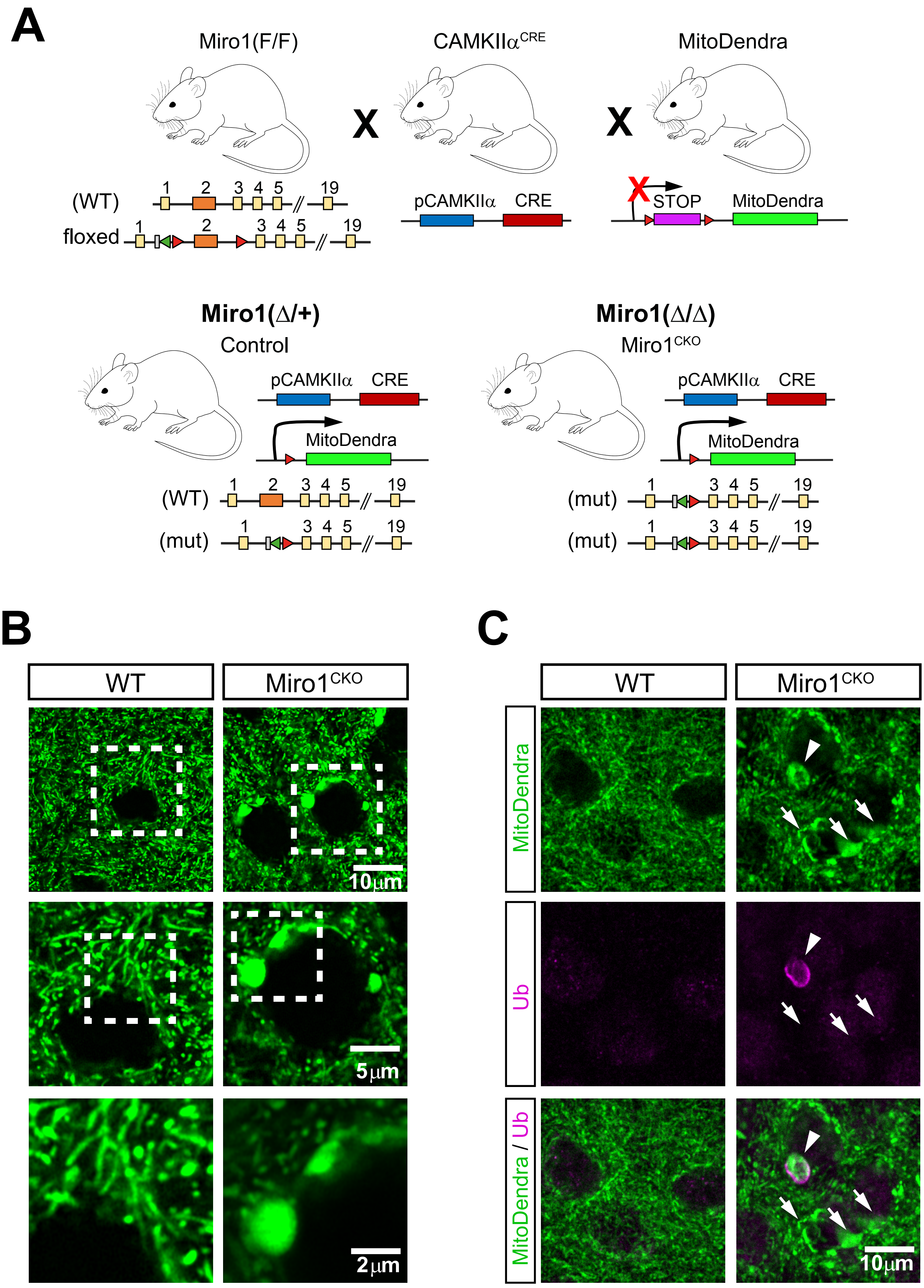

Appendix Figure S9
