# Supplementary figures and images for: Loss of neuronal Miro1 disrupts mitophagy and induces hyperactivation of the integrated stress response
Source: EMBO J. 2021 Jun 21;40(14):e100715. doi: 10.15252/embj.2018100715 (PMC8280823; doi:10.15252/embj.2018100715)

Appendix Figure S8C

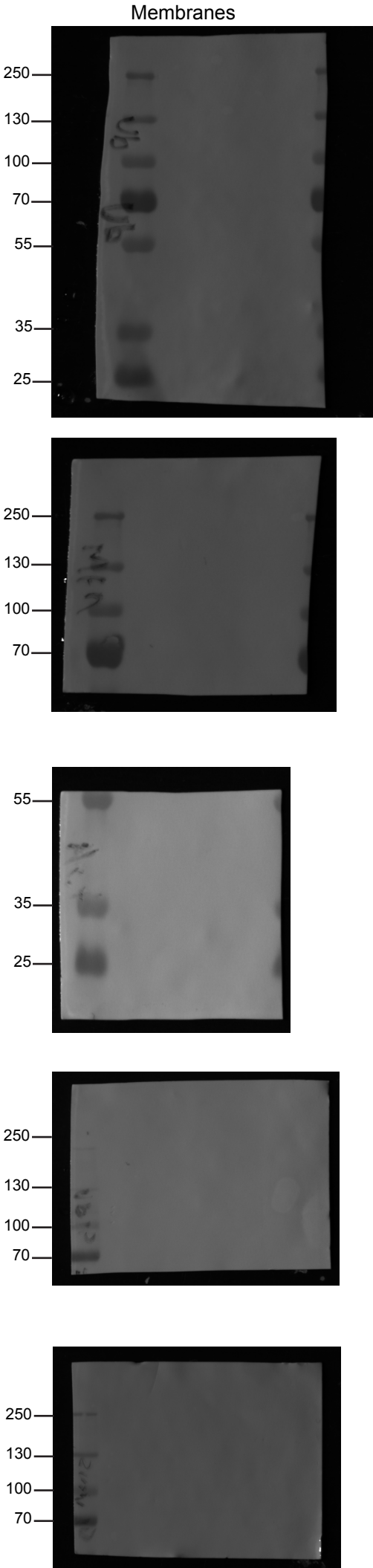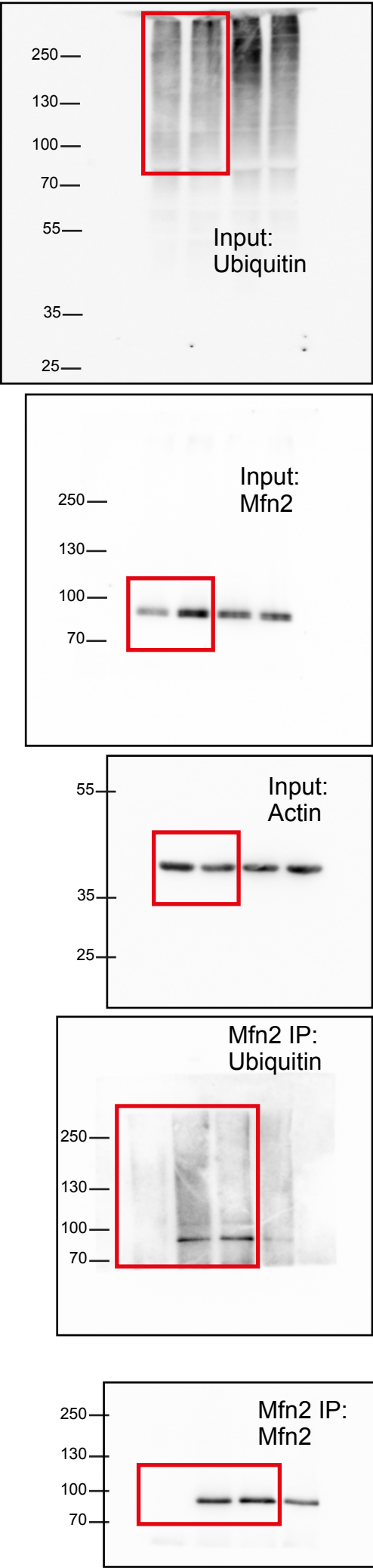

Supplement: Supplementary file 2 — Source Data for Appendix [file EMBJ-40-e100715-s006.zip › embj2018100715-sup-0006-SDataAppendixFigS8.pdf]

Figure 2C

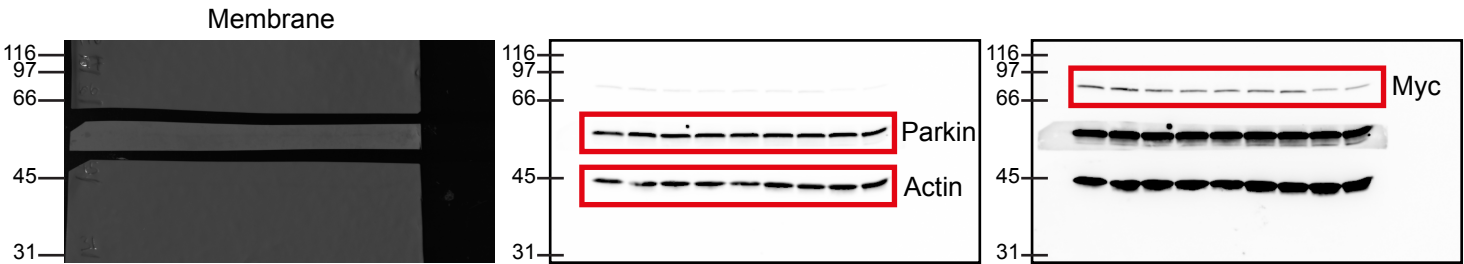

Figure 2D

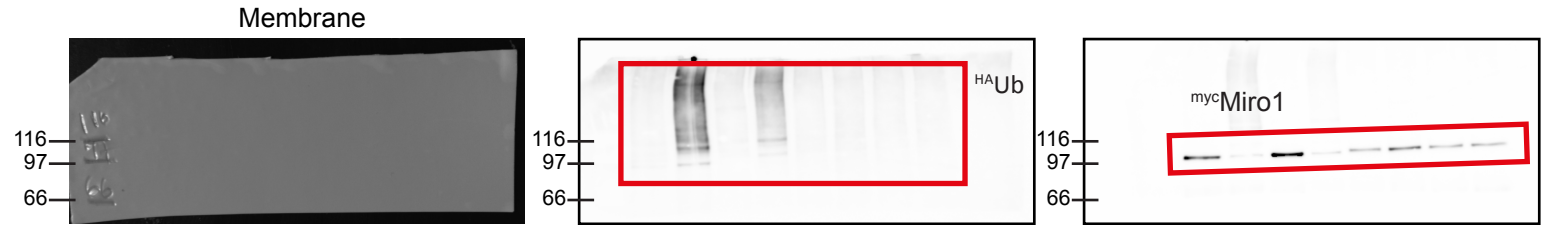

Figure 2F

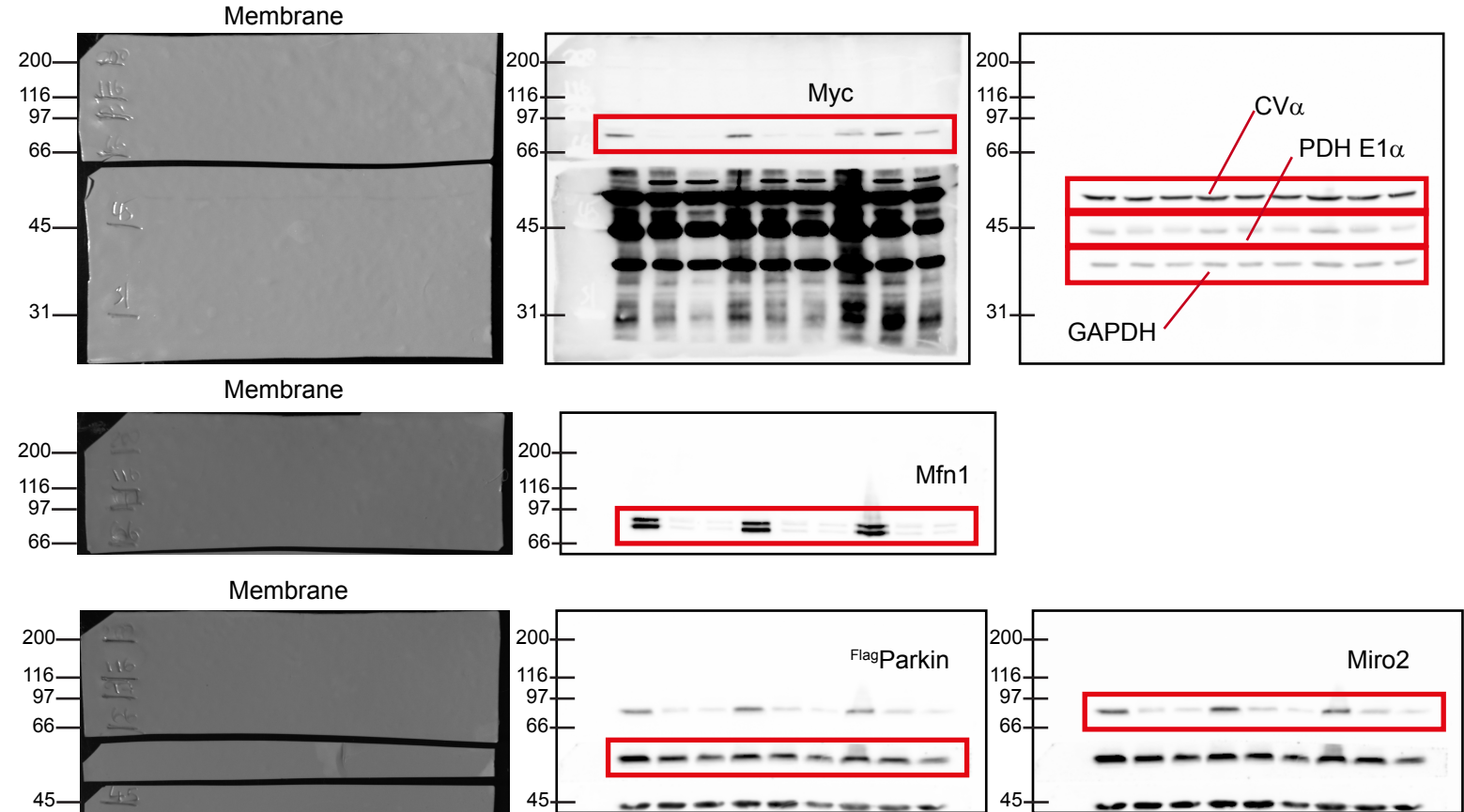

Supplement: Supplementary file 4 — Source Data for Figure 2 [file EMBJ-40-e100715-s005.pdf]

Figure 3D

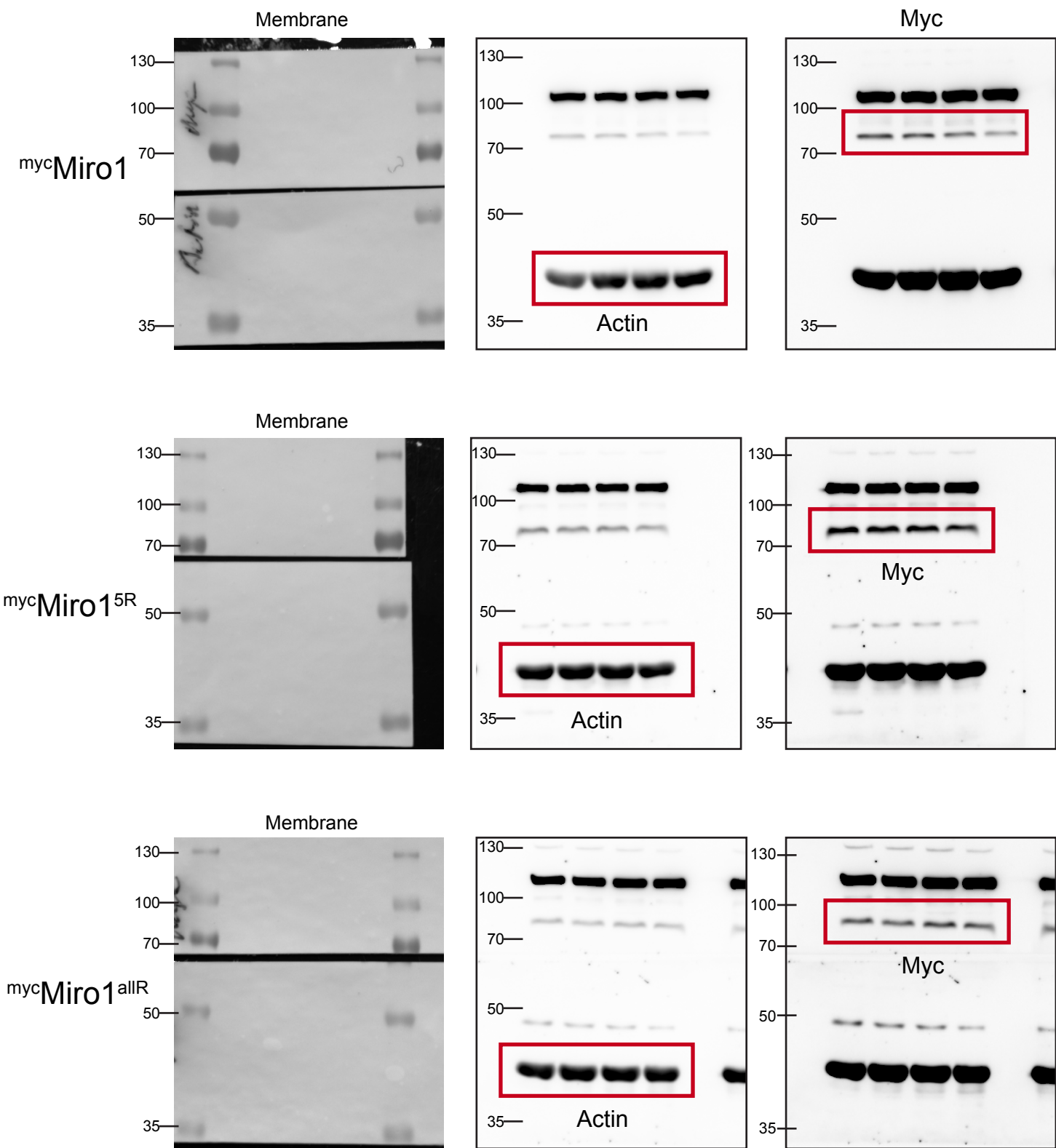

Supplement: Supplementary file 5 — Source Data for Figure 3 [file EMBJ-40-e100715-s002.pdf]

Figure 5A (4 months)

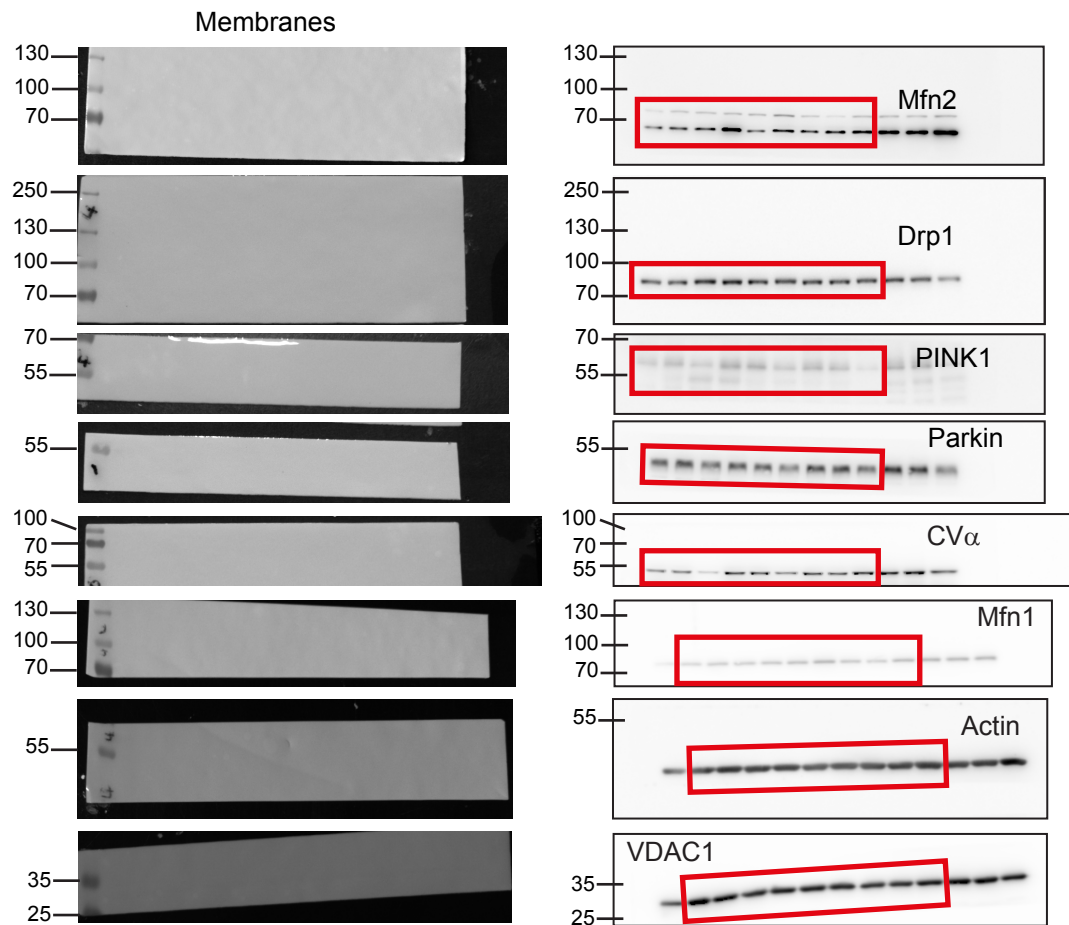

Figure 5A (12 months)

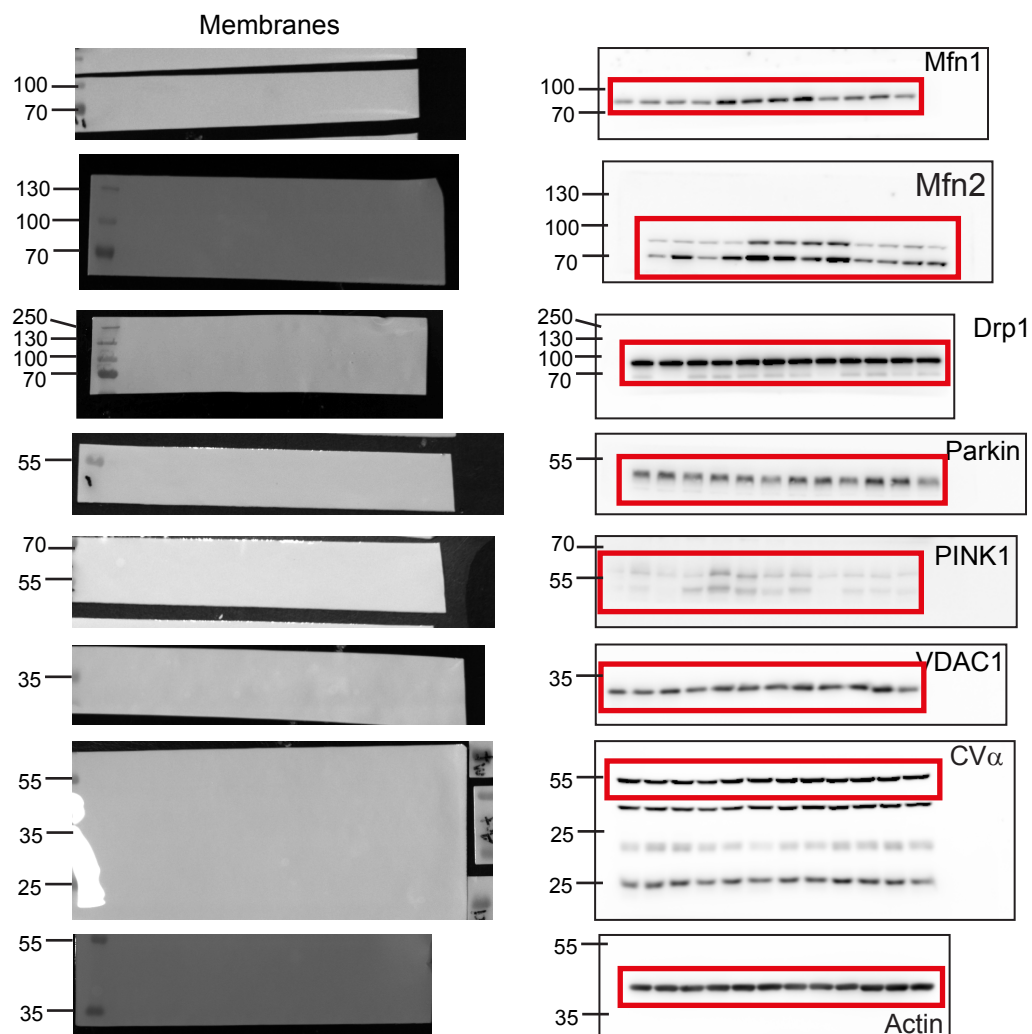

Supplement: Supplementary file 6 — Source Data for Figure 5 [file EMBJ-40-e100715-s001.pdf]

Figure 6A

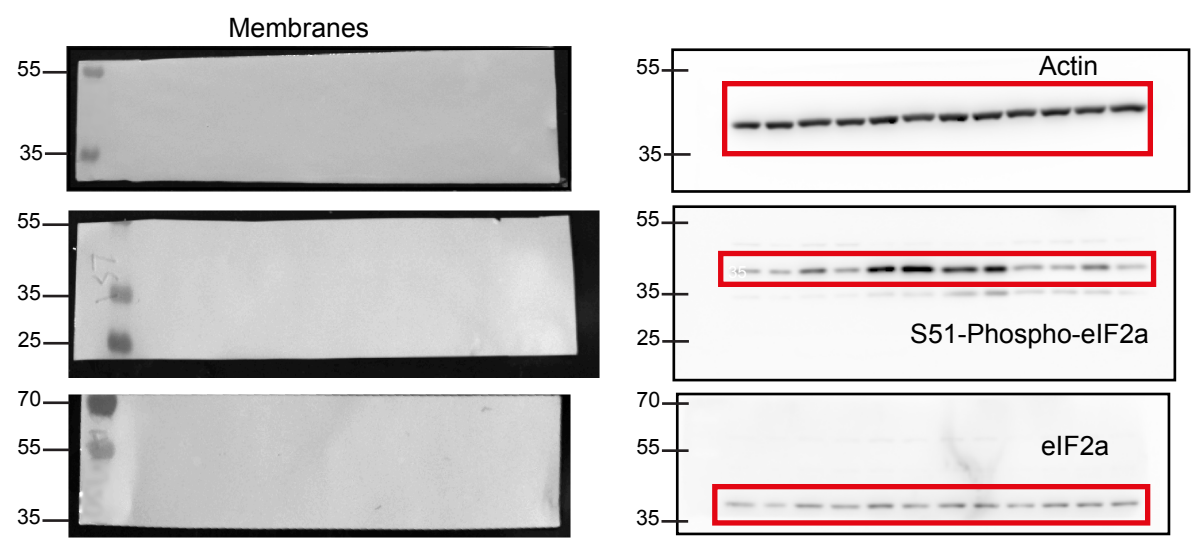

Supplement: Supplementary file 7 — Source Data for Figure 6 [file EMBJ-40-e100715-s004.pdf]
